# 

**Published:** 1988-02

**Authors:** 


Copyright © Clinical Press Ltd 1988.

### Contents

3 **Editorial**
5 **Medical Education and health care, is there a conflict?**
Charles Clarke
9 **Crohn's Disease—a misnomer?**
Michael Harmer
11 **Harvey Cushing and the secretion of the posterior pituitary into the C.S.F.**
T. Z. Aziz
13 **Computed Tomography of the internal auditory canals.**
D. J. Tawn, M. Snow, W. D. Jeans
17 **A Urine Flow Clinic**
M. E. Lucarotti, R. C. L. Fenelly, P. Abrams
21 **From our Correspondents—GERBIL strikes at fundamental academic freedoms, That's Life, Two Cornish 'Firsts', Work of Counsellors in G.P.**
23 **Correspondence—AIDS—Bristol Virologists reply to Dr Seale. G.P. and Hospital Medicine (*page 12*). Screening for allergy (*page 22*).**
25 **Surgical Club of South West England.**
28 **Critical extravaganzas—The Volvo 740 Turbo Estate, An evening with Beau Nash and Juliana Popjoy.**

